# Awareness on antibiotic prescription for endodontic cases among Dentists and Endodontists in Saudi Arabia

**DOI:** 10.6026/973206300200282

**Published:** 2024-03-31

**Authors:** Ahmed Ali Alelyani, Raid Abdullah Almnea, Rizwan Qureshi, Ziyad Mohammad Assiri, Mansour Hamad Alkorbi, Khaled Ahmed Almasabi, Siraj D.A.A Khan

**Affiliations:** 1 Restorative Department, Endodontic Division, Faculty of Dentistry, Najran University, Kingdom of Saudi Arabia; 2 Faculty of Dentistry, Najran University, Kingdom of Saudi Arabia; 3 Department of Preventive Dental Sciences, Faculty of Dentistry, Najran University, Kingdom of Saudi Arabia

**Keywords:** Endodontics, prescription, antibiotics, acute apical abscess

## Abstract

The knowledge of Dentists and Endodontists in Saudi Arabia regarding antibiotic prescriptions during and after endodontic treatment is of interest. A self-designed questionnaire survey was utilized to assess the knowledge of dentists across various cities in Saudi Arabia concerning antibiotic usage guidelines for endodontic purposes. A total of 391 participants were included in the study and the questionnaire was distributed through social platforms like WhatsApp, Instagram, Facebook Messenger and email. Results revealed that over 80% of participants acknowledged the need for antibiotics in cases of acute apical abscess with cellulitis, with amoxicillin being the most recommended antibiotic by dentists. Interestingly, there was no statistically significant difference in knowledge based on experience or graduation group. In conclusion, while participants demonstrated adequate knowledge about antibiotic prescriptions in endodontic cases, continued awareness of updated guidelines, including the WHO's Essential Medicines List (EML), guidelines by the European Society of Endodontology (ESE) and American Association Endodontists (AAE) is essential for optimal clinical practice.

## Background:

Antibiotics are substances derived from microbial sources or synthesized with similar properties, exhibiting antimicrobial effects in low concentrations to impede the growth or eradicate specific microorganisms. The purpose of antibiotic therapy is to assist the host's defense mechanisms in managing and eradicating temporarily overwhelming microorganisms [1]. Dentists often misuse antibiotics in various clinical situations. The primary approach for treating endodontic infections involves establishing and sustaining surgical drainage while eliminating the infection's root cause. Despite the valuable role of antibiotics, successful treatment in most cases can be achieved through mechanical and chemical cleaning of the root canal [2]. Clinicians have grappled with the persistent challenge of bacterial resistance to antimicrobials since the inception of these agents. This resistance stems from the inherent capability of bacterial species to develop resistance shortly after the introduction and widespread use of antibacterial agents [3]. Substantial evidence supports a noteworthy correlation between the surge in antimicrobial resistance and the utilization of antimicrobials. Bacteria isolated from regions with elevated antibiotic usage exhibit higher resistance levels compared to those from areas with lower antibiotic utilization [4]. One of the various benefits associated with antibiotics is their non-injurious impact on tissues. Additionally, antibiotics exhibit synergism, offering the potential to impact a broad spectrum of bacteria. They contribute to shortened sterilization duration and facilitate rapid healing [5]. However, it's essential to note that antibiotics do not alleviate odontogenic pain or swelling arising from teeth with symptomatic apical lesions when systemic involvement signs and symptoms are absent. The ineffectiveness of antibiotics in reaching the affected area is attributed to the lack of blood circulation in the root canal, particularly in necrotic teeth [6].

The systemic antibiotic prescription has become common in dental practice, increasing significantly in the last two decades. Dental antibiotic use represents 7-10% of global prescriptions for non-dental medical reasons [7]. Antibiotics are recommended based on European Society of Endodontology guidelines for pulp and periapical pathology. Prescription is warranted for systemic involvement (fever>38°C, malaise, lymphadenopathies, trismus), progressing infections (increased inflammation, cellulitis, osteomyelitis) and persistent infections in immunosuppressed individuals [8]. Not every endodontically involved tooth necessitates systemic antibacterial medication; effective management often involves pulp extirpation and thorough mechanical and chemical canal debridement. The use of antibiotics for immediate pain relief in acute pulpitis lacks proven benefits [9]. To improve this impending problem, scientific guidance based on scientific evidence was established by a committee of experts from the European Society of Endodontology (ESE) in 2018. The key feature of ESE is to emphasize the appropriate use of antibiotics in endodontics and the need to place more emphasis on the performance of root canal treatment exclusively. In particular, ESE places value on the risks associated with the inappropriate use of antibiotics and especially antibiotic resistance [8]. Palmer *et al.* found that 12.5% of general dental practitioners (GDPs) prescribed antibiotics for acute pulpitis, with 30.3% doing so due to time constraints and 47.3% in cases where a precise diagnosis was challenging. Amoxicillin was the most commonly prescribed antibiotic [10]. Another study revealed that 61.48% of GDPs preferred penicillin V for endodontic infections, while clindamycin (57.3%) and erythromycin (26.65%) were choices for those allergic to penicillin [11]. In Australia, a study showed a generally adequate level of antibiotic prescription knowledge among dentists, but there was a tendency towards over-prescription and a lack of awareness regarding adverse reactions, multi-resistant strains and bacterial endocarditis prophylaxis [12]. However, there is no data on Saudi dentists and endodontists regarding antibiotic prescription practices. Evaluating this information is crucial to identifying knowledge gaps, enabling the design of effective educational campaigns and addressing the issue of indiscriminate antibiotic use. Therefore, it is of interest to gather the knowledge of antibiotic prescription during endodontic treatment among dentists in Saudi Arabia.

## Materials and Methods:

## Study design:

This cross-sectional survey, employing a questionnaire-based approach, took place from August to December 2023. The survey aimed to assess the understanding of antibiotic prescriptions for endodontic cases among dentists and endodontics specialists. The questionnaire underwent initial scrutiny in a pilot study, with subsequent adjustments made to enhance its validity and reliability. The questionnaire was prepared based on Antibiotic prescription in Endodontic cases according to the ESE 2018 guidelines. Comprising two segments, Part A encompassed demographic details such as age, gender, work experience and year of graduation, while Part B focused on queries related to antibiotic prescription knowledge. These questions were presented in various formats, including multiple-choice questions and open-ended questions. The survey delved into practitioners' comprehension of the indications for prescribing antibiotics concerning systemic clinical signs associated with endodontic cases. Participants were queried about the necessity of antibiotics for specific clinical conditions, including acute pulpitis, acute apical abscess, chronic apical abscess with sinus tract and chronic apical periodontitis and their preferred treatment choices. Additionally, the survey explored various factors influencing antibiotic prescriptions.

## Sample size:

In total, 500 dentists were enlisted for this investigation and a questionnaire was distributed to them. Out of the total, 410 participants actively responded and submitted their completed questionnaires. To maintain data quality, questionnaires with less than 30% of questions answered were excluded, resulting in 391 questionnaires being available for subsequent analysis.

## Inclusion and exclusion criteria:

The inclusion criteria for this study involved general dentists and endo-dontists practicing in Saudi Arabia. Exclusions comprised practitioners who declined participation and those not involved in performing endodontic procedures. Participation was entirely voluntary, with participants retaining the right to withdraw from the study at any point without facing any consequences. The questionnaire's outset included a cover letter elucidating the survey's purpose and guaranteeing data confidentiality. Respondents signalled their consent to participate and were subsequently directed to complete the questionnaire. Personal identifiers such as names, emails, or any other private information were deliberately omitted from the collected data.

## Statistical analysis:

The data was analyzed using the SPSS software and the results were portrayed using descriptive measures. Correlations among various parameters were determined using χ^2^ tests.

## Ethical approval:

Approval for the ethical considerations of this study was granted by the Deanship of Research, Najran University under the reference number 202401-076-017905-040323.

## Results:

This study encompassed a total of 391 participants, consisting of 55 females (14.1%) and 336 males (85.9%) who were practicing dentistry. The majority of participants were distributed across Najran (94), Abha (68), Jeddah (58) and Riyadh (43). Regarding age distribution, 63.4% fell within the less than 30 years category, while 36.6% were aged over 30. In terms of professional experience, 35% had less than 3 years, 32.5% had 3-5 years and more than 5 years of experience. The graduation years 2016-2020 comprised the majority, accounting for 63.9% of the participants (Table 1). The majority of participants across various experience groups, including 123 (89.8%) with less than 3 years of experience, 102 (80.3%) with 3-5 years and 105 (31.8%) with over 5 years, there was a consensus that systemic antibiotics were necessary for patients diagnosed with acute apical abscess with cellulitis in endodontic cases. The preferred first-line antibiotics were Amoxicillin and Metronidazole, with 47.4%, 53.5% and 38.4% agreement from those with less than 3 years, 3-5 years and more than 5 years of experience, respectively. This choice was particularly favoured for patients in good health without documented allergies. Only 3 (2.2%) participants with less than 3 years of experience opted for clindamycin in such cases. In instances where patients were allergic to penicillin or amoxicillin, the majority across all experience groups recommended Clindamycin, comprising 118 (86.1%) with less than 3 years of experience, 110 (86.6%) with 3-5 years and 110 (86.6%) with more than 5 years of experience. Ciprofloxacin and a combination of clindamycin and ciprofloxacin were the least preferred antibiotics. For patients with a localized swelling and draining sinus, the majority across experience groups (56.2%, 55.9% and 66.9% for less than 3 years, 3-5 years and more than 5 years of experience, respectively) recommended a combination of antibiotics (Table 2). The recommended dosage which is chose by 50% participants was Penicillin VKa loading dose 1000 mg with a maintenance dose of 500 mg q4-6h 5-7 days followed by Amoxicillin loading dose 1000 mg with a maintenance dose of 500 mg q4-6h 5-7 days by 32% participants (Figure 1 - see PDF). A total of 36% participants were agreed that Patients with previous history of Infective Endocarditis need antibiotic prescription while only 5% agree that Patients with primary Endodontic lesions with secondary periodontal involvement need prescription of antibiotics (Figure 2 - see PDF). A large number of dentists across different graduation groups, including 57 (83.8%) from the 1st group (before 2015), 209 (83.6%) from the 2nd group (2016-2020) and 64 (87.7%) from the 3rd group (after 2020), there was consensus that systemic antibiotics were necessary for patients diagnosed with acute apical abscess with cellulitis in endodontic cases. Notably, 10.3% of participants from the 1st group opted for irreversible pulpitis and 9.6% from the 3rd group chose symptomatic apical periodontitis instead. Amoxicillin and Metronidazole were considered the primary antibiotics by most participants (55.9%, 58.8% and 42.5% from the 1st, 2nd and 3rd groups, respectively) for prescribing in endodontic cases requiring antibiotic treatment, particularly for patients in good health without documented allergies. Amoxicillin and Clavulanic acid emerged as the second most preferred antibiotics for these patients. For individuals allergic to penicillin or amoxicillin, Clindamycin was the top-recommended antibiotic by the majority of participants across all groups (58 (85.3%), 218 (87.2%) and 62 (85.0%) from the 1st, 2nd and 3rd groups, respectively). In cases involving localized swelling with a draining sinus, a combination of antibiotics was recommended by most participants (67.6%, 59.2% and 53.4% from each group, respectively) (Table 3). The recommended dosage and regimen for patients requiring prophylactic antibiotics in the context of endodontic treatment in an adult according to the 56% participants was Amoxicillin, oral route, 1g, 1 hour before procedure (Figure 3 - see PDF). For the patients who have allergy to amoxicillin in endodontic cases, the recommended dosage according to 63% participants was Clindamycin, oral route, 600 mg, 1h before procedure (Figure 4 - see PDF).

## Discussion:

This cross-sectional study, conducted through a questionnaire, aimed to evaluate the knowledge of antibiotic prescription practices among dentists and endodontists in Saudi Arabia during endodontic treatments. The findings revealed sufficient knowledge among participants regarding the prescription of antibiotics in endodontic therapy. It is worth noting that the present survey boasted a larger sample size (n=319) compared to a 2015 local study by Iqbal (n=157), demonstrating a notable strength [13]. Furthermore, the current study included participants from various cities and institutes in Saudi Arabia, distinguishing it from the single-institute study conducted by Iqbal *et al.* in 2015, which adds another layer of strength [13]. According to the British Society for Antimicrobial Chemotherapy, improper prescription of antibacterial drugs by dental practitioners significantly contributes to the emergence of drug-resistant strains. Factors such as inappropriate dosing, duration and prophylaxis play a role in the development of resistant strains [14]. Various factors, including the improper prescription of antibiotics by medical or dental practitioners, contribute to Antimicrobial Resistance (AMR) [8]. Once resistance occurs, reversing it becomes impossible, emphasizing the importance of minimizing the development of new resistant strains through the judicious use of antibiotics [15]. As per a systematic review conducted by James *et al.* antibiotics were deemed non-essential for providing relief in cases of irreversible pulpitis [16, 17]. Consistent with this, the research conducted by Vengidesh *et al.* identified a certain degree of antibiotic misuse, with prescriptions given for pain relief, reversible pulpitis, irreversible pulpitis and endodontic flare-ups (84.1%) [18]. In the current study, 330 (84.4%) dentists agreed that the prescription of antibiotics was deemed necessary in the condition of acute apical abscess with Cellulitis. Similarly, another study indicated that the highest percentage of antibiotic prescriptions was observed in cases of acute apical abscess with diffuse intraoral swelling, accompanied by fever and trismus (83.4%), as well as in cases of acute apical abscess with diffuse intra- and extra-oral swelling, fever and trismus (81%) [19]. These percentages align with the findings of a Brazilian survey, reporting figures of 88.1% and 90.1%, respectively [20]. To prevent antibiotic overuse, in 2018, the European Society of Endodontology (ESE) issued the most recent recommendations for prescribing practices related to endodontic infections and suggested that their members forward this information to dentists in their respective countries [8]. In the investigation conducted by Abdulhai *et al.* a significant majority of participants (75.3%) selected amoxicillin 500 mg, three times a day, as their primary choice for therapeutic antibiotics [19]. Similarly, in our study, Amoxicillin and Metronidazole were the first choice by 216 (55.2%) dentists. This percentage stands notably higher than what was reported in a prior local study (18.3% and 33.7%) and various international studies (34-47%) [13, 21, 22]. However, it is lower than the reported preferences of Brazilian endodontists (90.2%) and Spanish dental students (100%) [23]. According to ESE antibiotics guidelines in endodontics, Beta-lactam antibiotics (penicillin V and amoxicillin) are recommended for the treatment of endodontic infections. Recommended loading doses are 1000 mg of penicillin V administered orally followed by 500 mg every 4-6 h or 1000 mg of amoxicillin, with or without clavulanic acid, followed by 500 mg every 8h [8, 24]. Penicillin VK, being bactericidal, exhibits high effectiveness, low toxicity and cost-effectiveness [25]. Notably, penicillin has a relatively narrow spectrum, while amoxicillin boasts a broader spectrum of antibiotic activity [24, 25].

Amoxicillin, a synthetic improvement of the penicillin molecule, is easily absorbed with food and remains resistant to stomach acid damage [26]. The combination of amoxicillin with metronidazole has been recommended due to metronidazole's excellent activity against anaerobes [11]. In a separate study by Vengidesh *et al.* approximately 87% of participants chose amoxicillin as their primary drug, followed by metronidazole (11%), a pattern consistent with the findings of Maslamani *et al.* [18, 27]. In the research conducted by Mawra *et al.*, clindamycin emerged as a less commonly prescribed option [28]. Clindamycin was also the least chosen antibiotic in the present study. These findings align with studies by Fahad *et al.* and Jain A *et al.*, where clindamycin was infrequently prescribed, while amoxicillin was the more prevalent choice [29, 30]. A combination of antibiotics is favored by over 70% of dentists and amoxicillin + clavulanic acid is one such combination recommended for severe oral infections and situations where resistant species are suspected, unresponsive to standard endodontic procedures [31]. This percentage is lower than that reported by Turkish dentists (90.3%) but higher than figures from Iqbal *et al.* (45.2%), Rodriguez-Nunez *et al.* (42%), Martin-Jimenez *et al.* (53%) and Bolfoni *et al.* (26%) [15, 20, 21, 23, 32]. Moreover, 86.5% of participants in the current study chose clindamycin for the patients who exhibit an allergy to penicillin. This figure is almost the same as the one reported by Martin-Jimenez *et al.* (99%) but higher than the range observed in previous studies, which varied from 4.4% to 65% [13, 25, 32, 33- 34]. The 2017 AAE guidelines emphasize the recommendation to prescribe antibiotics for diabetic patients with poor glycemic control in the context of antibiotic prophylaxis [35]. The ESE position statement on antibiotic use in endodontics further advocates' antibiotic prophylaxis for medically compromised patients experiencing acute apical abscess, cases with systemic involvement, progressive infections, replantation of avulsed permanent teeth and soft tissue trauma [31]. The ESE also indicates prophylaxis before endodontic procedures for patients with other conditions, including impaired immunologic function, prosthetic joint replacement, high-dose jaw irradiation and intravenous bisphosphonates [8]. The initiation of endodontic infections is characterized by a rapid onset with a brief duration, typically resolving within 3-7 days or less if the underlying cause is adequately treated or removed [36]. A reported study indicates that 52.4% of participants would prescribe antibiotics for cases of acute apical abscess with localized intraoral swelling and pain [19]. This practice raises concerns as the necrotic pulp system lacks effective circulation and the primary treatment for such cases involves incision and drainage, followed by root canal treatment (RCT) or extraction of the affected tooth to eliminate the source of infection [36]. Furthermore, 22.2% of respondents expressed the inclination to use antibiotics for treating necrotic pulp with chronic apical periodontitis featuring a fistula but no pain [19]. This percentage aligns with the findings of Rodriguez-Nunez *et al.* (21.4%, 2009) but contradicts the observations of Segura-Egea *et al.* (60%), Martin-Jimenez *et al.* (38%) and Iqbal (46.6%) [13, 23, 32, 33].

## Conclusion:

Data shows that participants had an adequate knowledge about the prescription of antibiotics in endodontic cases but it highlighted the tendency among dentists, to prescribe antibiotics, deviating from established guidelines. It is imperative to place greater emphasis on instilling a proper antibiotic prescription approach at the undergraduate level. Additionally, dental practitioners should stay abreast of recent guidelines for antibiotic prescription, including the WHO's Essential Medicines List (EML) and the AWaRe classification. Continuous Dental Education (CDE) programs can play a pivotal role in this regard, ensuring practitioners are well-informed. Following precise endodontic diagnosis and treatment protocols is essential to mitigate endodontic flare-ups, subsequently diminishing the necessity for antibiotics. Educating patients about the potential adverse effects of self-prescribing antibiotics is crucial in promoting responsible antibiotic use.

## Figures and Tables

**Table 1:** Demographic Details of Participants

**Gender**		
Female	55	14.1
Male	336	85.9
Total	391	100
**Area of Practice**		
Abha	68	17.4
Al Madina	33	8.4
Al-Ahsa	2	0.5
Aljouf	3	0.8
Bisha	3	0.8
Buraidah	2	0.5
Dammam	7	1.8
Hail	5	1.3
Jazan	23	5.9
Jeddah	58	14.8
Khamis Mushait	5	1.3
Khobar	4	1
Makkah	18	4.6
Najran	94	24
Riyadh	43	11
Sharurah	11	2.8
Tabuk	7	1.8
Taif	5	1.3
Total	391	100
**Age**		
Less than 30 years	248	63.4
More than 30 years	143	36.6
Total	391	100
**Experience**		
Less than 3 years	137	35
3 to 5 years	127	32.5
More than 5 years	127	32.5
Total	391	100
**Graduation Group**		
Before 2015	68	17.4
2016-2020	250	63.9
After 2020	73	18.7
Total	391	100

**Table 2:** Correlation between working experience and awareness

**Response**		**Experience**			**Total**	**Chi - square**	**p - value**
		**Less than 3 years**	**3-5 years**	**More than 5 years**			
In which conditions is the prescription of systemic antibiotics deemed necessary for patients with an endodontic diagnosis	Acute Apical Abscess with Cellulitis	123 89.8%	102 80.3%	105 31.8%	330 84.4%	19.098	0.014
	Chronic Apical Abscess	1 0.7%	0 0%	0 0%	1 0.3%		
	Irreversible Pulpitis	1 0.7%	10 7.9%	15 11.8%	26 6.6%		
	Necrotic Pulp with Asymptomatic Apical Periodontitis	5 3.6%	5 3.9%	4 3.1%	14 3.6%		
	Symptomatic Apical Periodontitis	7 1.8%	10 2.6%	3 2.4%	20 5.1%		
What is the recommended primary / first line antibiotic for prescription in cases of endodontically related conditions necessitating antibiotic treatment, for patients who are in good health and have no documented allergies	Amoxicillin and Clavulanic acid	42 30.7%	35 27.6%	28 22%	105 26.9%	14.068	0.08
	Amoxicillin and Metronidazole	65 47.4%	68 53.5%	83 38.4%	216 55.2%		
	Clindamycin	3 2.2%	0 0%	0 0%	3 0.8%		
	Erythromycin	5 3.6%	3 2.4%	3 2.4%	11 2.8%		
	Penicillin VK	22 16.1%	21 16.5%	13 10.2%	56 14.3%		
Which antibiotic is the optimal choice when a patient exhibits an allergy to penicillin Or amoxicillin in the context of endodontic treatment?	Ciprofloxacin	5 3.6%	2 1.6%	2 1.6%	9 2.3%	11.457	0.489
	Clarithromycin	5 3.6%	7 5.5%	4 3.1%	16 4.1%		
	Clindamycin	118 86.1%	110 86.6%	110 86.6%	338 86.5%		
	Clindamycin, Ciprofloxacin	4 2.9%	3 2.4%	2 1.6%	9 2.3%		
	Minocycline	3 2.2%	1 0.8%	7 5.5%	11 2.8%		
	Minocycline, Clindamycin	2 1.5%	4 3.1%	2 1.6%	8 2%		
Under what circumstances should a combination of antibiotics, such as amoxicillin and Metronidazole, be considered in endodontic treatment?	Patients having a localised swelling with draining sinus	77 56.2%	71 55.9%	85 66.9%	233 59.6%	5.296	0.506
	Patients showing no improvement in symptoms with previous medication of Amoxicillin alone	41 29.9%	36 28.3%	27 21.3%	104 26.6%		
	Patients with allergy to Penicillin VK	7 5.1%	10 7.9%	6 4.7%	23 5.9%		
	Patients with sharp and shooting pain and tooth tender on percussion	12 8.8%	10 7.9%	9 7.1%	31 7.9%		
In which clinical scenario antibiotic prescription is a consideration for preventing Post-treatment flare-ups / Post treatment pain in endodontic cases?	Acute Apical Abscess with localised fluctuant swellings, elevated body temperature >38°C, malaise, lymphadenopathy, trismus	99 72.3%	97 76.4%	105 82.7%	301 77%	10.734	0.097
	Irreversible Pulpitis with Condensing Osteitis	14 10.2%	15 11.8%	6 4.7%	35 9%		
	Necrotic pulp with Periapical Radiolucency	21 15.3%	9 7.1%	13 10.2%	43 11%		
	Patients with tooth fractures, concussion, subluxation, luxation injuries and extrusion injury	3 2.2%	6 4.7%	3 2.4%	12 3.1%		
Is antibiotic prescription advisable in cases where a radiograph reveals a substantial periapical radiolucency associated with a draining sinus/ purulent discharge in Endodontic patients?	No, not indicated in healthy patients	71 51.8%	64 50.4%	73 57.5%	208 53.2%	4.382	0.357
	Yes, in retreatment cases to prevent post-operative pain	10 7.3%	6 4.7%	3 2.4%	19 4.9%		
	Yes, for better treatment outcomes	56 40.9%	57 44.9%	51 40.2%	164 41.9%		
Should antibiotics be prescribed for patients who present with severe pain in response to hot and cold stimuli, along with clinical signs of percussion tenderness, to	No, not indicated in healthy patients	86 62.8%	76 59.8%	80 63%	242 61.9%	9.109	0.058
	Yes, in retreatment cases to prevent	31	17	18	66		

**Table 3:** Correlation between graduation group and awareness

**Response**		**Graduation group**			**Total**	**Chi - square**	**p - value**
		**Before 2015**	**2016-2020**	**After 2020**			
1.In which conditions is the prescription of systemic antibiotics deemed necessary for patients with an endodontic diagnosis of	Acute Apical Abscess with Cellulitis	57 83.8%	209 83.6%	64 87.7%	330 84.4%	12.177	0.143
	Chronic Apical Abscess	0 0%	1 0.4%	0 0%	1 0.3%		
	Irreversible Pulpitis	7 10.3%	19 7.6%	0 0%	26 6.6%		
	Necrotic Pulp with Asymptomatic Apical Periodontitis	3 4.4%	9 3.6%	2 2.7%	14 3.6%		
	Symptomatic Apical Periodontitis	1 1.5%	12 4.8%	7 9.6%	20 5.1%		
What is the recommended primary / first line antibiotic for prescription in cases of endodontically related conditions necessitating antibiotic treatment, for patients who are in good health and have no documented allergies	Amoxicillin and Clavulanic acid	18 26.5%	60 24%	27 37%	105 26.9%	15.114	0.057
	Amoxicillin and Metronidazole	38 55.9%	147 58.8%	31 42.5%	216 55.2%		
	Clindamycin	0 0%	1 0.4%	2 2.7%	3 0.8%		
	Erythromycin	3 4.4%	4 1%	4 5.5%	11 2.8%		
	Penicillin VK	9 13.2%	38 15.2%	9 12.3%	56 14.3%		
Which antibiotic is the optimal choice when a patient exhibits an allergy to penicillin or amoxicillin in the context of endodontic treatment?	Ciprofloxacin	1 1.5%	7 2.8%	1 1.4%	9 2.3%	8.081	0.779
	Clarithromycin	2 2.9%	11 4.4%	3 4.1%	16 4.1%		
	Clindamycin	58 85.3%	218 87.2%	62 85%	338 86.5%		
	Clindamycin, Ciprofloxacin	2 2.9%	5 2%	2 2.7%	9 2.3%		
	Minocycline	3 4.4%	5 2%	3 4.1%	11 2.8%		
	Minocycline, Clindamycin	2 0.5%	4 1.6%	2 2.7%	8 2%		
Under what circumstances should a combination of antibiotics, such as Amoxicillin and metronidazole, be considered in endodontic treatment?	Patients having a localised swelling with draining sinus	46 67.6%	148 59.2%	39 53.4%	233 59.6%	6.709	0.349
	Patients showing no improvement in symptoms with previous medication of Amoxicillin alone	16 23.5%	68 27.2%	20 27.4%	104 26.6%		
	Patients with allergy to Penicillin VK	2 2.9%	17 6.8%	4 5.5%	23 5.9%		
	Patients with sharp and shooting pain and tooth tender on percussion	4 5.9%	17 6.8%	10 13.7%	31 7.9%		
In which clinical scenario antibiotic prescription is a consideration for Preventing post-treatment flare-ups / Post treatment pain in endodontic cases?	Acute Apical Abscess with localised fluctuant swellings, elevated body temperature >38°C, malaise, lymphadenopathy, trismu	58 85.3%	200 80%	43 58.9%	301 77%	24.359	0
	Irreversible Pulpitis with Condensing Osteitis	5 7.4%	20 8%	10 13.7%	35 9%		
	Necrotic pulp with Periapical Radiolucency	5 7.4%	20 8%	18 24.7%	43 11%		
	Patients with tooth fractures, concussion, subluxation, luxation injuries and extrusion injury	0 0%	10 4%	2 2.7%	12 3.1%		
Is antibiotic prescription advisable in cases where a radiograph reveals a Substantial periapical radiolucency associated with a draining sinus/purulent discharge in endodontic patients?	No, not indicated in healthy patients	37 54.4%	137 54.8%	34 46.6%	208 53.2%	3.678	0.451
	Yes, in retreatment cases to prevent post operative pain	1 1.5%	13 5.2%	5 6.8%	19 4.9%		
	Yes, for better treatment outcomes	30 44.1%	100 40%	34 46.6%	164 41.9%		
Should antibiotics be prescribed for patients who present with severe pain in response to hot and cold stimuli, along with clinical signs of percussion	No, not indicated in healthy patients	39 57.4%	156 64.5%	47 64.4%	242 61.9%	0.869	0.929
Tenderness, to expedite symptom relief in endodontic cases?	Yes, in retreatment cases to prevent post operative pain Yes, in retreatment cases to prevent post operative pain	13 19.1%	41 16.4%	12 16.4%	66 16.9%		
	Yes, for better treatment outcomes	16 23.5%	53 21.2%	14 19.2%	83 21.2%		
